# Reconstruction of ancient homeobox gene linkages inferred from a new high-quality assembly of the Hong Kong oyster (*Magallana hongkongensis*) genome

**DOI:** 10.1186/s12864-020-07027-6

**Published:** 2020-10-15

**Authors:** Yiqian Li, Wenyan Nong, Tobias Baril, Ho Yin Yip, Thomas Swale, Alexander Hayward, David E. K. Ferrier, Jerome H. L. Hui

**Affiliations:** 1 School of Life Sciences, Simon F.S. Li Marine Science Laboratory, State Key Laboratory of Agrobiotechnology, The Chinese University of Hong Kong, Shatin, Hong Kong; 2 Department of Conservation and Ecology, Penryn Campus, University of Exeter, Exeter, UK; 3 Dovetail Genomics, Scotts Valley, USA; 4 The Scottish Oceans Institute, Gatty Martine Laboratory, University of St. Andrews, St Andrews, UK

## Abstract

**Background:**

Homeobox-containing genes encode crucial transcription factors involved in animal, plant and fungal development, and changes to homeobox genes have been linked to the evolution of novel body plans and morphologies. In animals, some homeobox genes are clustered together in the genome, either as remnants from ancestral genomic arrangements, or due to coordinated gene regulation. Consequently, analyses of homeobox gene organization across animal phylogeny provide important insights into the evolution of genome organization and developmental gene control, and their interaction. However, homeobox gene organization remains to be fully elucidated in several key animal ancestors, including those of molluscs, lophotrochozoans and bilaterians.

**Results:**

Here, we present a high-quality chromosome-level genome assembly of the Hong Kong oyster, *Magallana hongkongensis* (2n = 20), for which 93.2% of the genomic sequences are contained on 10 pseudomolecules (~ 758 Mb, scaffold N50 = 72.3 Mb). Our genome assembly was scaffolded using Hi-C reads*,* facilitating a larger scaffold size compared to the recently published *M. hongkongensis* genome of Peng et al. (Mol Ecol Resources, 2020), which was scaffolded using the *Crassostrea gigas* assembly. A total of 46,963 predicted gene models (45,308 protein coding genes) were incorporated in our genome, and genome completeness estimated by BUSCO was 94.6%. Homeobox gene linkages were analysed in detail relative to available data for other mollusc lineages.

**Conclusions:**

The analyses performed in this study and the accompanying genome sequence provide important genetic resources for this economically and culturally valuable oyster species, and offer a platform to improve understanding of animal biology and evolution more generally. Transposable element content is comparable to that found in other mollusc species, contrary to the conclusion of another recent analysis. Also, our chromosome-level assembly allows the inference of ancient gene linkages (synteny) for the homeobox-containing genes, even though a number of the homeobox gene clusters, like the Hox/ParaHox clusters, are undergoing dispersal in molluscs such as this oyster.

## Background

Homeobox-containing genes encode transcription factors that are widely employed in animal, plant and fungi development, and are frequent foci for the evolution of diverse body plans and morphologies. Homeoboxes generally encode a 60–63 amino acid domain known as the homeodomain [21, 33]. A notable feature of animal homeobox genes is that they often exist in genomic clusters, due to either coordinated gene regulation or possibly phylogenetic inertia (i.e. lack of dispersal via genomic rearrangements following a common origin via tandem duplication). Homeobox clusters include: the ANTP-class (Hox, ParaHox, NK, Mega-homeobox, SuperHox), the PRD-class (HRO), TALE-class (Irx), and SINE-class (SIX), all of which may have descended from a Giga-cluster state [10, 25, 26, 29, 34, 52, 68]. The best-known homeobox cluster is that of the Hox genes in the ANTP-class, where sequential expression of genes from along the cluster patterns development both spatially and temporally [22]. Taxonomically wide comparisons between high quality genome assemblies provide vital data to better understand these cases of homeobox gene clustering and linkage, and facilitate a deeper understanding of the evolutionary mechanisms and events involved.

Bilaterians can largely be divided into three major clades: the lophotrochozoans, ecdysozoans and deuterostomes, which together comprise the majority of animal species [49]. However, most of our understanding of homeobox clustering originates from studies that have focused on ecdysozoans and deuterostomes. Lophotrochozoans include annelids and molluscs, and recent years have seen increasing numbers of genomes sequenced within the Mollusca (Table 1).

**Table 1:** Genome statistics for mollusc genomes

Class		Speices	Family	Accession number	Assembly size	Number of scaffolds	Contig N50	Scaffold N50	BUSCOs	No.Proteins	Reference
**Bivalvia**	**	*Anadara broughtonii*	Arcidae	PRJNA521075	884,566,040	1026	1,797,717	44,995,656	97.70%	24,045	[4]
		*Argopecten irradians concentricus*	Pectinidae	GCA_004382765.1	874,784,041	82,208	63,725	1,246,717	91.00%	25,979	[45]
		*Argopecten irradians irradians*	Pectinidae	GCA_004382745.1	835,595,382	111,436	78,654	1,533,165	91.00%	26,777	[45]
		*Bathymodiolus platifrons*	Mytilidae	GCA_002080005.1	1,659,280,971	65,664	12,602	343,373	94.40%	33,584	[77]
		*Crassostrea gigas*	Ostreidae	GCF_000297895.1	557,735,934	7659	31,239	401,685	94.40%	46,748	[95]
		*Dreissena rostriformis*	Dreissenidae	GCA_007657795.1	1,241,703,712	18,514	45,905	131,390	83.30%	37,681	[12]
	**	*Cyclina sinensis*	Veneridae	GCA_012932295.1	903,119,975	187	2,587,078	46,470,132	92.70%	27,564	[90]
		*Limnoperna fortunei*	Mytilidae	GCA_003130415.1	1,673,223,206	20,580	32,203	309,123	81.90%	60,717	[85]
		*Mizuhopecten yessoensis*	Pectinidae	GCF_002113885.1	987,588,634	82,659	65,014	803,631	95.50%	41,567	[88]
		*Modiolus philippinarum*	Mytilidae	GCA_002080025.1	2,629,649,654	74,575	19,700	100,161	84.20%	36,549	[77]
		*Pinctada fucata*	Pteriidae	PRJDB2628	815,303,973	29,306	1629	167,048	91.10%	31,477	[82]
	**	*Pinctada fucata martensii*	Pteriidae	GCA_002216045.1	990,984,031	5039	21,518	59,032,463	86.30%	/	[20]
	**	*Pecten maximus*	Pectinidae	GCA_902652985.1	918,306,378	3983	1,258,799	44,824,366	95.50%	67,741	[39]
	**	*Panopea generosa*	Hiatellidae	GCA_902825435.1	942,353,201	18	14,495	57,743,597	66.70%	/	/
		*Ruditapes philippinarum*	Veneridae	GCA_009026015.1	1,123,164,463	30,670	29,238	345,005	91.00%	27,652	[94]
		*Saccostrea glomerata*	Ostreidae	GCA_003671525.1	788,118,542	10,107	39,800	804,232	92.10%	29,738	[58]
	**	*Sinonovacula constricta*	Pharidae	GCA_007844125.1	1,220,848,272	2450	976,936	65,929,677	91.92%	28,594	[65]
	**	*Crassostrea virginica*	Ostreidae	GCF_002022765.2	684,741,128	11	1,971,208	75,944,018	94.40%	60,213	/
	**	*Crassostrea hongkongensis*	Ostreidae	CNP0000529	610,039,375	660	2,576,225	55,627,392	95.80%	25,675	[61]
	**	***Magallana hongkongensis***	Ostreidae	**WFKH00000000**	**757,928,205**	**11,926**	**49,472**	**72,332,161**	**94.60%**	**45,867**	**This study**
		*Mytilus coruscus*	Mytilidae	GCA_011752425.1	1,903,825,920	10,484	817,337	898,347	96.44%	42,684	[44]
		*Venustaconcha ellipsiformis*	Unionidae	GCA_003401595.1	1,590,012,607	371,427	2813	6657	68.00%	/	[66]
**Cephalopoda**		*Euprymna scolopes*	Sepiolidae	GCA_004765925.1	5,280,013,996	59,146	3558	3,549,550	97.00%	29,089	[6]
		*Octopus bimaculoides*	Octopodidae	GCF_001194135.1	2,338,188,782	151,674	5532	475,182	86.50%	23,994	[2]
	**	*Octopus sinensis*	Octopodidae	GCA_006345805.1	2,719,151,902	13,516	490,217	105,892,736	50.00%	/	[44]
		*Architeuthis dux*	Architeuthidae	GCA_006491835.1	3,155,388,500	7276	9000	5,478,336	85.50%	51,225	[23]
**Gastropoda**	**	*Achatina fulica*	Achatinidae	PRJNA511624	1,855,883,074	1010	721,038	59,589,303	91.70%	23,726	[30]
	**	*Achatina immaculata*	Achatinidae	GCA_009760885.1	1,653,153,977	563	3,802,429	56,367,627	96.27%	28,702	[45]
		*Anentome helena*	Nassariidae	WUUA000000000.1	1,720,191,841	2,637,315	56,088	2,075,175	/	/	/
		*Biomphalaria glabrata*	Planorbidae	GCF_000457365.1	916,388,084	331,401	7298	48,059	87.70%	36,675	[3]
		*Elysia chlorotica*	Plakobranchidae	GCA_003991915.1	557,480,303	9989	441,954	30,474	93.30%	24,980	[11]
		*Haliotis discus*	Haliotidae	PRJNA317403	1,865,475,499	80,032	41,000	200,099	90.70%	29,449	[56]
		*Haliotis laevigata*	Haliotidae	GCA_008038995.1	1,762,655,385	105,411	3353	81,233	84.56%	55,164	[8]
		*Haliotis rufescens*	Haliotidae	GCA_003343065.1	1,498,703,277	8371	283,651	1,895,871	95.10%	57,785	[51]
		*Haliotis rubra*	Haliotidae	GCA_003918875.1	1,378,265,264	2854	1,177,711	1,227,833	94.60%	44,137	[28]
		*Lanistes nyassanus*	Ampullariidae	GCA_004794575.1	507,389,202	17,149	25,785	317,839	96.30%	20,938	[78]
		*Lottia gigantea*	Lottiidae	GCF_000327385.1	359,505,668	4469	96,027	1,870,055	94.40%	23,822	[76]
		*Limacina bulimoides*	Limacinidae	SWLX000000000.1	2,901,939,372	3,735,750	884	893	30.30%	/	[19]
		*Marisa cornuarietis*	Ampullariidae	GCA_004794655.1	535,287,142	659	4,359,112	4,359,112	98.20%	23,827	[78]
	**	*Pomacea canaliculata*	Ampullariidae	GCF_003073045.1	440,159,624	24	1,072,857	31,531,291	94.40%	40,391	[47]
		*Pomacea maculata*	Ampullariidae	GCA_004794325.1	432,264,763	3908	75,997	375,864	96.40%	23,475	[78]
	**	*Chrysomallon squamiferum*	Peltospiridae	GCA_012295275.1	404,615,235	22	1,880,000	30,197,626	96.60%	16,917	[79]
		*Radix auricularia*	Lymnaeidae	GCA_002072015.1	909,764,068	4823	24,354	578,730	93.40%	17,338	[80]

**Chromosome level assembly

True oysters are important on both ecological and economic levels. In the marine ecosystem, oysters serve as keystone species fulfilling roles in both water filtration, and creating bottom substrate for other organisms on the oyster reef. In addition, they are also a source of high-quality protein for a range of wildlife, including many birds, and for human consumption. Oyster farming has a long history and can be traced back to the early Roman Empire (500 BC) in Europe [27], and the Han dynasty (206 BC-220 AD) in Asia (FAO FISHERIES TECHNICAL PAPER 427 Aquaculture Development in China The Role of Public Sector Policies). Bivalves more generally are a highly important food source, with global production of marine bivalves for human consumption exceeding 15 million tonnes per year between 2010 to 2015, equating to ~ 14% of total global marine production [86]. Within marine bivalve shellfish catches, ~ 89% originate from aquaculture, and China contributes 85% of total world production and hence holds considerable food security importance in this sector [86].

The best known extant true oysters include: the European flat oyster (*Ostrea edulis*) in Europe; the Eastern oyster (*Crassostrea virginica*) and the Olympia oyster (*Ostrea lurida*) in North America; the Pacific oyster (*Magallana gigas* - previously *Crassostrea gigas*) which is native to the Pacific coast of Asia, but has been introduced to Australia, Europe, and North America; and the Sydney rock oyster (*Saccostrea glomerata*) endemic to Australia and New Zealand. Previous studies have reported the genomes of several true oysters. The Pacific oyster has a reported genome size of between 545 and 637 Mb [95]. Meanwhile, the Sydney rock oyster (*S. glomerata*) has a reported genome size of 784 Mb [58]. In addition, the genome of the pearl oyster (*Pinctada fucata*) has a reported genome size of 990 Mb [82], but is not a species of true oyster, instead belonging to the family Pteriidae.

The Hong Kong oyster (*Magallana hongkongensis*, previously known as *Crassostrea hongkongensis*, Lam and Morton [42, 71]) is a species of true oyster cultivated in the mouth of the Pearl River Delta, southern China, and in surrounding coastal regions of Guangdong Province [43]. The species is found on intertidal and subtidal rocks, and oyster farms along Deep Bay (‘*Hau Hoi Wan*’ in Cantonese) [43]. In Hong Kong, the mudflats at *Lau Fau Shan* in Deep Bay are currently the only area involved in cultivation of *M. hongkongensis*, with a history in this activity dating back hundreds of years to when Hong Kong was just a fishing village. Despite the scientific, ecological, cultural, and nutritional importance of *M. hongkongensis*, a high-quality genome sequence has been lacking until very recently (see [61]), hindering scientifically-informed aquaculture science, and wider scientific understanding of the species. Moreover, both the sustainability of the Hong Kong oyster, and its harvest as a food commodity, are currently threatened by pollution. Heavy metal contamination is a particular problem, which holds challenges for exploitation of oysters as a food source (e.g. [89, 91]). Ocean acidification is an emerging threat to the conservation and sustainability of the oyster, especially due to the vulnerability of the thin-shelled spat [54]. Meanwhile, the presence of antibiotic resistant bacteria in oysters is a growing problem with significant potential negative health consequences [92]. Taking into account the above challenges, the production and availability of high-quality genomic resources for this species is particularly important.

This study provides a new chromosome-level assembly of *M. hongkongensis* constructed on sequencing results from a single individual. A recent study also provided a chromosome-level assembly of the same species, but an important difference is that reads were anchored to another species *Crassostrea gigas* to achieve higher sequence continuity as indicated by scaffold N50 [61]. Given the considerable estimated divergence time between *M. hongkongensis* and *C. gigas* (~ 26 MYA, range: 23.47–28.78 MYA, corresponding to more than four times the evolutionary distance between human and chimp )[41], this approach is problematic for at least two reasons: 1) many gene order inferences are likely to be inaccurate, and, 2) it was not possible to anchor many scaffolds to the supposed 10 pseudomolecules. We also provide detailed comparative analyses of transposable elements and homeobox genes in the *M. hongkongensis* genome as a means to assess generalities of genome content and organization, given: (i) the important role of transposable elements in genome size and rearrangements during evolution, and, (ii) the importance of homeobox genes as markers of chromosome-level linkage evolution or synteny (e.g. SuperHox, Mega-cluster, and Giga-cluster). We find that transposable element content is much more in-line with the prevalences inferred for other mollusc species, in contrast to the recent analyses of Peng et al. [61]. Also, we detect remnants of many homeobox gene clusters and ancient linkages, consistent with hypotheses on the ancestral existence of Hox/ParaHox, NK, SuperHox, Mega- and Giga-cluster arrangements.

## Results and discussion

### Data analyses

**Fig. 1:**
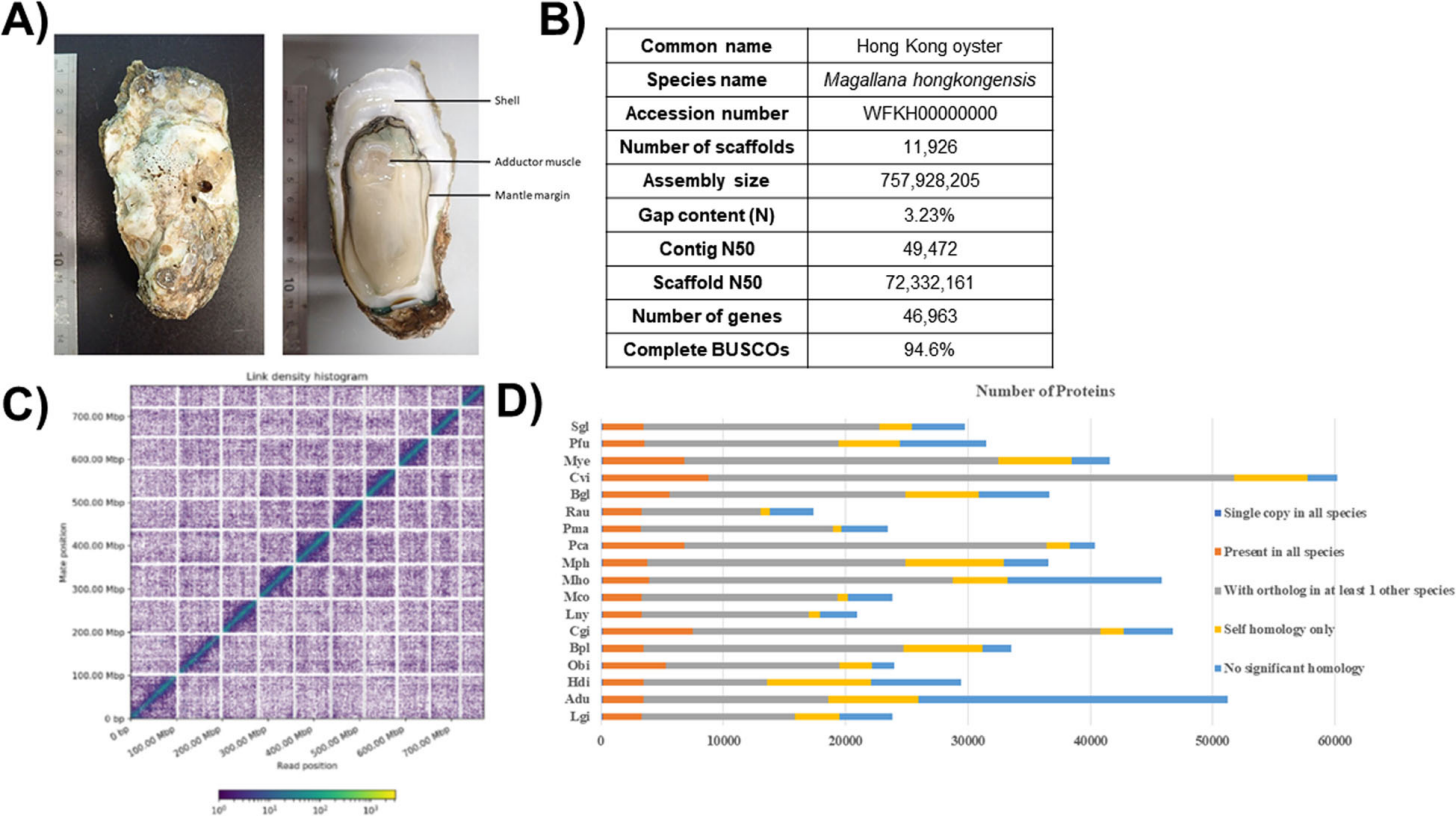
**a** A picture of the Hong Kong oyster *Magallana hongkongensis*; **b** Table summarising the statistics of the genome assembly for *M. hongkongensis*; **c** Hi-C information for *M. hongkongensis*. The x- and y- axes illustrate the mapping positions of the first and second read respectively in each read pair, grouped into bins. The colour of each square illustrates the number of read pairs within that bin. White vertical and black horizontal lines have been added to show the borders between scaffolds. Scaffolds less than 1 Mb are excluded; **d** Orthologous proteins comparison between all mollusc genomes. Abbreviations: Adu: *Architeuthis dux*; Bgl: *Biomphalaria glabrata*; Bpl: *Bathymodiolus platifrons*; Cgi: *Crassostrea gigas*; Cvi: *Crassostrea virginica*; Hdi: *Haliotis discus*; Lgi: *Lottia gigantea*; Lny: *Lanistes nyassanus*; Mco: *Marisa cornuarietis*; Mho: *Magallana hongkongensis*; Mph: *Modiolus philippinarum*; Mye: *Mizuhopecten yessoensis*; Obi: *Octopus bimaculoides*; Pca: *Pomacea canaliculata*; Pfu: *Pinctada fucata*; Pma: *Pomacea maculata*; Rau: *Radix auricularia*; Sgl: *Saccostrea glomerata*

**Fig. 2:**
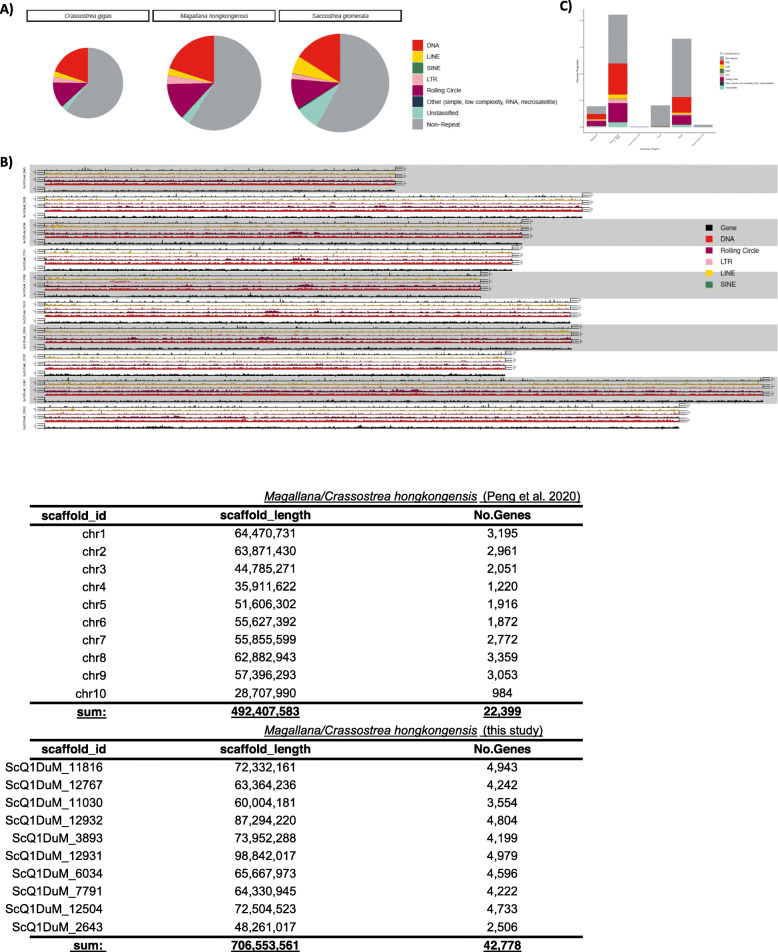
Comparison of genome assemblies between this study and [61]

This high-quality *M. hongkongensis* genome assembly and annotation has a comparable genome size (757 Mb) and number of predicted protein coding genes (45,308 generating 45,867 proteins) relative to other sequenced mollusc genomes (Table 1, Fig. 1d), and a comparable BUSCO coverage (94.6%, Metazoa Odb10) [75] relative to other published bivalve genomes (Fig. 1b, Table 1). Comparison between the genome assemblies from this and the previous Peng et al. [61] assembly is shown in Fig. 2. Considering the higher percentage of sequences contained on the ten pseudomolecules, similar gene orders based on syntenic analyses, and the method of construction for the genome assembly reported here, it is reasonable to conclude the information provided in this study is more reliable. It also has a high level of sequence continuity similar to the best standard in other published mollusc genomes (i.e. scaffold N50 = 72.3 Mb, Fig. 1b, Table 1), highlighting the high quality of this genome assembly. The chromosome number of *M. hongkongensis* has previously been determined (2n = 20, [55]), and we have found that 93.22% of the genomic sequences are contained on 10 pseudomolecules (Fig. 1c), indicating the first bona-fide chromosome-level genome assembly for *M. hongkongensis* made without recourse to linkage data from another species.

### Analyses of transposable elements

**Fig. 3:**
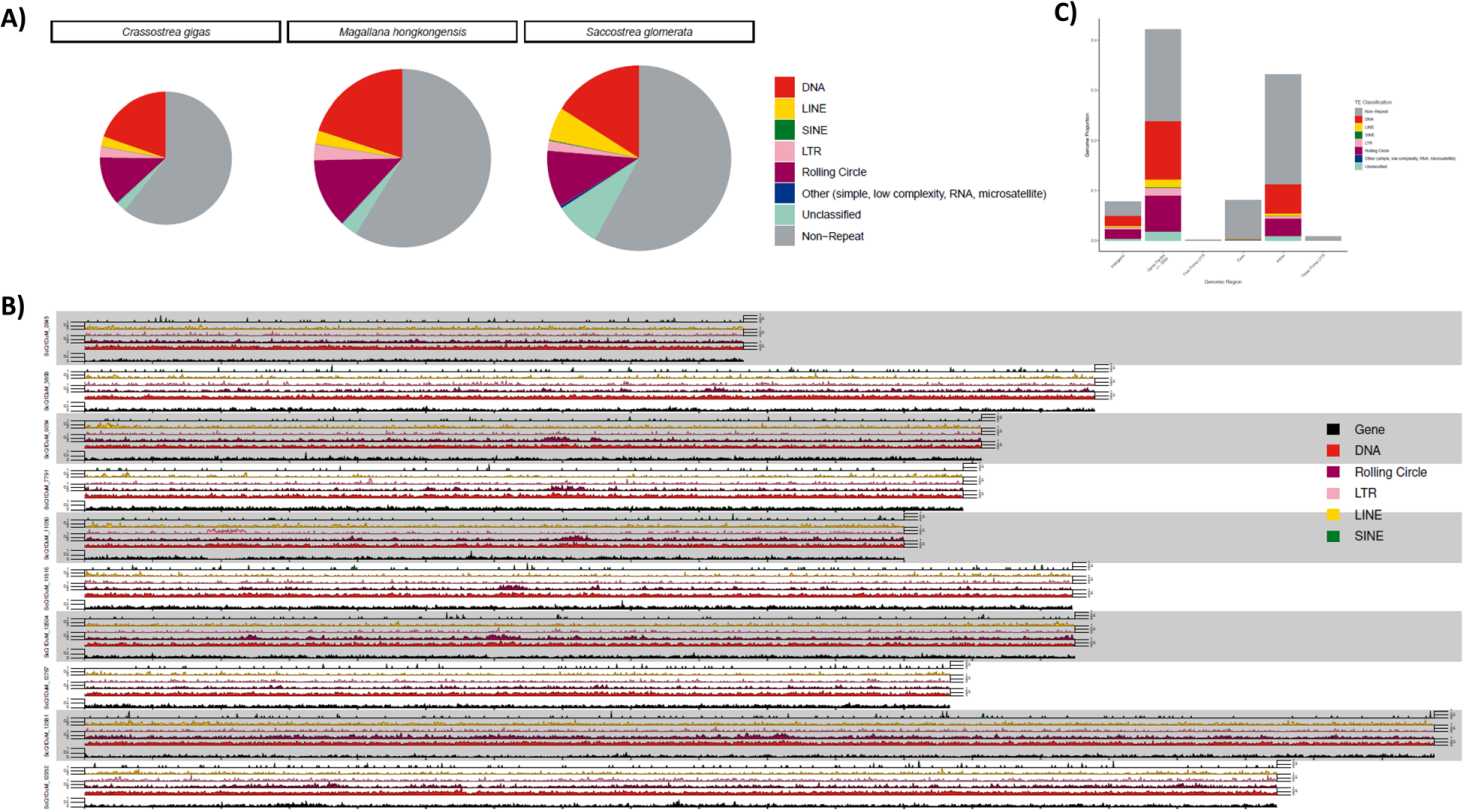
Analyses of transposable elements in oyster genomes. **a** Pie charts in proportion to genome size, indicating the proportions of repeat types present in the genome of each oyster species. The charts show a high contribution from transposable elements, and especially DNA transposons. **b** Overview of the insertional context of repeats in the *Magallana hongkongensis* genome, indicating that these are split relatively equally between genic and integenic locations. **c** Summary of transposable element activity in each oyster genome. These plots suggest a gradual increase in activity, peaking relatively recently, that was driven by increased proliferation of DNA and rolling circle transposons

Eukaryotic genomes contain a substantial proportion of repetitive DNA, and repeats are frequenty an important contributor to overall genome size [16]. The genomes of true oysters are no exception, with a repeat content of ~ 40% for available species in the Ostreidae (Supplementary information S2, Fig. 3a). To provide a comparative context, we analysed the repeat content of the newly sequenced Hong Kong oyster, *Magallana hongkongensis*, alongside the other available true oyster genomes, the Pacific oyster, *Crassostrea gigas* [95], and the Sydney rock oyster, *Saccostrea glomerata* [58]. We applied a conservative repeat annotation approach, focusing on high scoring matches, and discarding very short fragments unlikely to represent real repeat sequence (see Methods). We found that total repeat content is remarkably constant among available true oyster genomes, with variation spanning just 2.69% of total genome size (Table 2, Fig. 3a). The highest repeat content was identified in the Hong Kong oyster (41.12%), followed by the Sydney rock oyster (40.53%), and the Pacific oyster (38.43%) (Supplementary Information S2, Fig. 3a). Our results are similar to those published in the genome papers of the Sydney Rock oyster (45.03%) [58], and the Pacific oyster (36.1%) [95], but slightly more conservative given the more stringent approach undertaken in our pipeline (see Methods).

**Table 2:** Comparison of repeats in oyster genomes

Repeat Class	Pacific oyster (*Crassostrea gigas*)			Sydney rock oyster (*Saccostrea glomerata*)			Hong Kong oyster (*Magallana hongkongensis)* (This study)			Hong Kong oyster (*Magallana hongkongensis)* ([61]: CNGB Accession: CNP0000529)		
	No. elements	Total Length (Mb)	Percentage sequence (%)	No. elements	Total Length (Mb)	Percentage sequence (%)	No. elements	Total Length (Mb)	Percentage sequence (%)	No. elements	Total Length (Mb)	Percentage sequence (%)
**Retroelement**	37,163	28,544,850	5.06	120,895	58.86	7.46	52,102	40.18	5.3	42,786	34.49	5.65
*SINE*	1223	236,226	0.04	3279	1.11	0.14	1264	0.30	0.04	1105	0.24	0.04
*LINE*	21,075	13,539,249	2.40	102,086	44.00	5.58	28,141	18.21	2.40	24,710	16.73	2.74
*LTR element*	14,865	14,769,375	2.62	15,530	13.75	1.74	22,697	21.67	2.86	16,971	17.52	2.87
**DNA transposon**	267,568	108,351,108	19.18	309,542	118.32	15.01	376,197	151.49	19.99	262,645	129.15	21.17
**Rolling-circle**	96,923	65,056,555	11.52	158,233	69.40	8.81	138,281	95.49	12.60	105,724	89.47	14.67
**Unclassified**	24,313	13,835,725	2.45	133,418	61.23	8.81	40,091	23.05	3.04	22,365	14.97	2.45
**Other**	4829	1,288,331	0.22	10,362	3.48	0.44	5561	1.43	0.19	14,840	3.35	0.55
Small RNA	0	0	0.00	0	0.00	0.00	0	0.00	0.00	0	0.00	0.00
Satellites	991	252,370	0.04	686	0.16	0.02	614	0.19	0.03	634	0.15	0.02
Simple repeats	3795	1,029,211	0.18	9666	3.32	0.42	4902	1.24	0.16	14,150	3.20	0.53
Low complexity	43	6750	0.00	10	0	0.00	45	0.00	0.00	56	0.00	0.00
**Total repeats**	430,796	217,076,569	38.43	732,450	311.29	40.53	612,232	311.64	41.12	448,360	271.43	44.49

The genome size of the Hong Kong oyster (~ 758 Mb) is similar to that of the Sydney rock oyster (~ 788 Mb), but the Pacific oyster has a considerably smaller genome (~ 565 Mb). Both the Sydney rock oyster and Hong Kong oyster have a repeat content of ~ 311 Mb, while the Pacific oyster has a repeat content of just 217 Mb (Fig. 1, Supplementary information S2). Thus, repeats appear to have played a role in the expansion of genome size in the Hong Kong oyster and Syndney rock oyster. However, there appears to have been a corresponding non-repeat contribution to the increase in genome size also, since the non-repeat proportion of the genome remains relatively constant across all three genomes (58.9–61.6%).

We find that the vast majority of transposable elements (TEs) identified in the Hong Kong oyster, and in true oyster genomes more widely, are DNA elements (DNA transposons and Rolling-circle elements), which account for 23.8–32.6% of total genomic content, with the Hong Kong oyster representing the upper end of this scale (Supplementary information S2, Fig. 3a). Retroelements (SINEs, LINEs, and LTR elements) make up a much smaller proportion of the genome (5.06–7.46%), with SINEs particularly poorly represented in oyster genomes (0.04–0.14%) (Table 2, Fig. 3a).

Given the high quality of our Hong Kong oyster genome assembly and accompanying gene annotation, we analysed the distribution of TEs across the genome to examine patterns of host gene-TE association. At a coarse level, TEs of each major category are distributed relatively evenly across the entire host genome (Fig. 3b). However, at a fine scale, TEs are disproportionately represented in regions flanking genes (defined here as plus or minus 20 kb either side of a gene) and in introns, with the most common elements (i.e. DNA TEs, including rolling circle elements) driving this pattern (Fig. 3c). As expected, TE activity has been largely excluded from exons, thereby protecting host gene function.

Repeat landscape plots (Supplementary information S3), suggest that repeat activity in the Hong Kong oyster has trailed off recently following a sustained gradual increase in activity. This pattern is similar across all three true oyster species, with patterns in TE activity primarily driven by the proliferation of DNA elements, including rolling-circle elements (Supplementary information S3). Only the Sydney rock oyster shows evidence of a notable proliferation in retroelements (i.e. LINE elements of the penelope group, Supplementary information S3), which is reflected in the higher proportion of these elements in the genome (5.58%, Supplementary information S2).

Collectively, the observed patterns suggest that true oyster genomes have been strongly influenced by the activity of TEs, and particularly by DNA transposons. As more true oyster genomes become available, detailed analyses of the processes driving these patterns will become possible, and the Ostreidae represents a promising group for the study of host-transposon interactions, and especially DNA elements.

We note considerable discrepancies between the results of our repeat annotation and corresponding results reported in a recently released genome assembly of the Hong Kong oyster, particularly in relation to proportions of identified LTR elements [61]. Consequently, we downloaded and analysed the assembly of Peng et al. [61], in an attempt to replicate their findings. Using our comprehensive TE annotation pipeline incorporating well tested and verified urrent capproaches, we identify an LTR abundance of 2.88% in the assembly of Peng et al. [61] (Class: LTR, Supplementary information S2), very close to the result for our assembly of 2.86%, but at odds with the figure of 19% reported in Peng et al. [61]. Additionally, we find a reduction in the abundance of LINE, DNA, and Unclassified elements, along with a reduction in sequences classed as “Other”, compared to the study of Peng et al. [61].

Several explanations exist for the disparity between our results and those of Peng et al. [61]. Firstly, Peng et al. [61] used dated versions of RepeatMasker (v4.0.7) and the associated RepBase library (v21.12), lacking important upgrades (e.g. v4.0.8: updated libraries for RepBase, including 4500 new families; v4.0.9: updated support for combined TE consensus sequence libraries with Dfam HMM profiles, improving TE identification and classification. At the time of release, Dfam support added 6235 TE family sequences). Meanwhile, several known problems exist for older versions of RepeatMasker, such as classification instabilities, where consecutive runs on the same assembly can lead to the same TE being assigned to different repeat names and class/family attributes (https://github.com/rmhubley/RepeatMasker/issues/64). Secondly, Peng et al. [61] use LTR_STRUC to identify LTR elements, a dated program released in 2003 [53]. Attempts to obtain this software to replicate results were unsuccessful, given the requirement for an obsolete version of Windows and broken download links. However, a recent study benchmarking different LTR identification methods noted the high False Discovery Rate (FDR) of LTR_STRUC, due to“imprecisely defined sequence boundaries of LTR candidates [57]. Given this, we used LTR_FINDER [93] and LTRharvest [24], followed by LTRdigest [81] to classify putative LTR elements. Whilst also relatively old programs, these are widely recognised as leading methods, and the combination of LTR_FINDER and LTRharvest is noted to achieve high performance when benchmarked against other methods [57]. Thirdly, the difference in LTR abundance between a standard bare RepeatMasker run (often the default adopted in genome assembly projects for repeat masking and repeat analysis) and our pipeline is just 1.6% of total genome assembly size. We find that RepeatMasker performs well in identifying LTR TEs in genomes, where the increase in abundance following LTR-specific programs often comes from re-defining LTR boundaries and interiors, rather than from the identification of new LTR elements completely missed by RepeatMasker. Given this, it is highly unlikely that RepeatMasker should miss LTR elements making up ~ 16% of the total genome assembly, as reported by Peng et al. [61]. Fourthly, published analyses of closely related oyster species agree more closely with our findings: Total repeat content: Sydney rock oyster = 45% [58], Pacific oyster = 36% [95], Hong Kong oyster (this study) = 41%, Hong Kong oyster [61] = 57%; LTR TE content: Sydney rock oyster = 1.74% [58], Pacific oyster = 2.5% [95], Hong Kong oyster (this study) = 2.86%, Hong Kong oyster [61] = 19%. Collectively, our inability to reproduce the results of Peng et al. [61], discrepancies with other published studies, and methodological issues, suggest problems with the repeat analysis of Peng et al. [61], and the utility of our results as an alternative reference.

### Homeobox genes

In the *M. hongkongensis* genome, a total of 135 homeobox genes were identified using reciprocal BLAST and gene phylogeny construction (Supplementary information S4, 5, 6), which is very similar to the 136 homeobox genes identified in the Pacific oyster *Crassostrea gigas* [69].

**Fig. 4:**
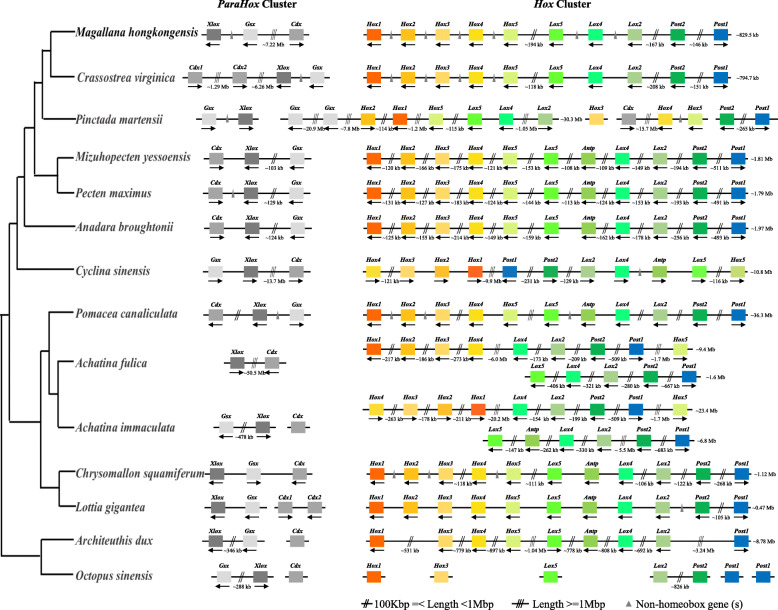
Hox and ParaHox gene clusters in different mollusc genomes

**Fig. 5:**
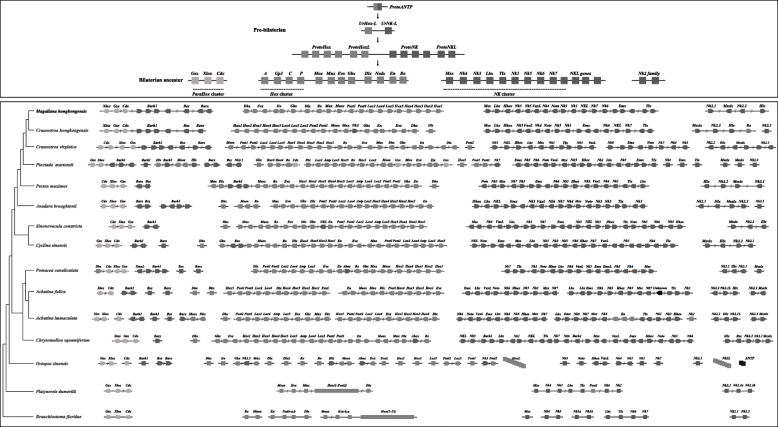
ANTP-class homeobox genes in 13 mollusc genomes. Diagonal lines mean large distance between genes (with chromosome linkage). The two lines indicates the distance is more than 100 kb and less than 1 Mb, the three lines indicates the distance is over 1 Mb. Ur- and Proto- HoxL designate Hox cluster-linked genes (i.e. non-Hox homeobox genes linked to the Hox cluster genes), whilst Ur- and Proto- NKL designate NK cluster-linked genes (i.e. non-NK homeobox genes linked ot the NK cluster genes)

**Fig. 6:**
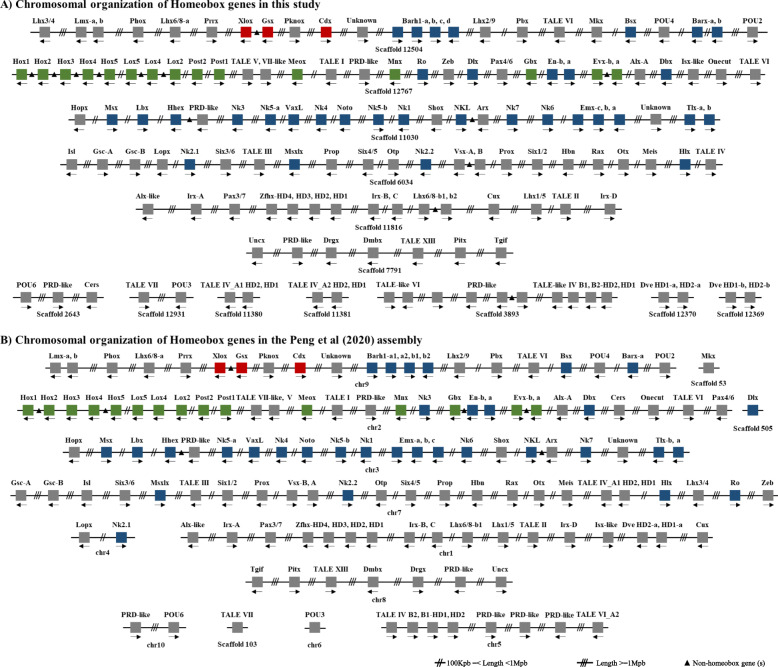
Homeobox gene organisations in the oyster *M. hongkongensis* genome assembly generated in this study (upper panel) and a recently published assembly, [61] (lower panel). Black triangles denote that there are intervening non-homeobox gene(s)

The ANTP-class of homeobox genes represents the biggest class of homeobox genes in animals and includes the Hox, ParaHox, and NK clusters, which are of great importance in understanding animal evolution and development [33]. In both the oyster *C. gigas* and the scallop *Pinctada fucata*, Hox gene clusters are distributed over distinct scaffolds, and certain Hox genes appear to have been lost during evolution [82, 95]. Given that both the scallop *Mizuhopecten yessoensis* and the limpet *Lottia gigantea* contain intact Hox clusters without loss of any lophotrochozoan Hox genes [76, 88], it is generally believed that the last common ancestor of oysters experienced Hox gene cluster reorganisation. This contrasts greatly to the situation that we uncover in *M. hongkongensis*, where a Hox cluster with a full complement of genes is revealed (Fig. 4, 5, 6). However, it is notable that non-homeobox genes are present between Hox genes, and thus it should be considered to be a ‘disorganized’ Hox cluster [22]. In addition, in both the Hox clusters of *L. gigantea* and *M. hongkongensis*, the posterior gene *Post1* is transcribed in a different orientation to the rest of the Hox cluster genes (Fig. 4, 5). This implies that a *Post1* inversion had already occurred in the last common ancestor of molluscs, and was one of the first stages of the mollusc Hox cluster becoming ‘Disorganized’.

In *M. hongkongensis*, the three ParaHox genes (*Gsx, Xlox,* and *Cdx*) are linked on the same scaffold (Fig. 4, 5, 6). Careful analyses of genomic organization across available mollusc genome assemblies revealed that in the majority of species the ParaHox cluster has broken apart. However, for *Pinctada fucata*, *Bathymodiolus platifrons, Mizuhopecten yessoensis* and *Marisa cornuarietis* the three ParaHox genes are still relatively closely linked, but often with one or more intervening non-homeobox gene(s) (Fig. 4, 5). This implies that functional constraints that keep this cluster intact in animals like chordates are not operating in most, and maybe all, of the sampled mollusc lineages. This may be a distinctive feature of molluscs, since dispersal of the ParaHox genes would be expected to be more extensive if the loss of clustering constraints was more ancient. It will be interesting to see what impact, if any, these rearrangements have had on the regulation and expression of mollusc ParaHox genes in future work.

The NK gene cluster is compact in insects but disrupted in vertebrates [17, 26, 36, 46]. This pattern contrasts with that of the Hox and ParaHox gene clusters, which are generally compact in vertebrates [22, 26]. In *M. hongkongensis* the remnants of an NK cluster can be seen, with NK genes dispersed along the same chromosome among non-homeobox genes. This example of an atomized NK cluster, which has not progressed to the level of genes dispersed across distinct chromosomes, involves the *Msx, Lbx, Hhex, NK3, NK5, Vax, NK4, Noto, NK5, NK1, Vent-like, NK7, NK6, Emx* and *Tlx* genes on Scaffold 11,030 in *M. hongkongensis* (Fig. 6). This retention of these NK genes on the same chromosome is perhaps analogous to the situation found in drosophilids, in which NK cluster genes have secondarily reassembled into clustered arrangements during evolution [17], however, the small chromosome number in these flies complicates the comparison. It will be intriguing to see, with further chromosome-level assemblies of other bivalves or even molluscs, whether a case of secondary cluster formation similar to that of drosophilids is also found, and if so, what effect this has on gene regulation and expression. Our data is in line with the hypothesis that there are different selection forces and functional constraints acting on Hox, ParaHox, and NK clusters in different animal clades, including the lophotrochozoans, and that descriptions of Hox/ParaHox and NK ‘clusters’ are often be an oversimplification that overlooks intriguing organizational diversity, with important connotations for understanding of the regulation of developmental genes.

A principal guiding hypothesis for the evolutionary origins of Hox, ParaHox, and NK clusters is that there was a clustered array, the so-called “Megacluster”, that in-turn contained the “SuperHox” cluster, linking certain ANTP-class homeobox genes very early in animal evolution, at least prior to the origin of the bilaterians ([10, 15, 26, 29, 34, 68]). From the mapping of certain ANTP-class homeobox genes in the polychaete *Platynereis dumerillii*, the latest consensus is that the Hox genes, ParaHox genes, NK genes, and NK2 family genes were located on four chromosomes in the bilaterian ancestor [26, 33, 34] (Fig. 5). With our chromosome-level assembly of the genome of *M. hongkongensis*, we observe that the Hox, ParaHox, NK genes, and NK2 family genes are located on just four scaffolds, as hypothesized for the bilaterian ancestor from work on *Platynereis* and amphioxus (Figs. 5 and 6). Consequently, this implies an extremely low level of inter-chromosomal rearrangement on oyster, polychaete and chordate lineages relative to the bilaterian ancestor, making these useful taxa with which to reconstruct the chromosome-level organization of this ancient ancestor’s genome.

Another class of homeobox genes that have been frequently studied in the context of understanding animal evolution is the PRD-class. The PRD-class HRO cluster contains homeobox genes *Hbn/ArxL-Rax-Otp* and has been detected in comparisons of cnidarians, protostomes and deuterostomes [18, 26, 52]. In *M. hongkongensis*, a dispersed but syntenic grouping of *Gsc-Prop-Otp-Vsx-Hbx-Rax-Otx* has been recovered on scaffold 6034 (Fig. 6). Considering that *Gsc* and *Otx* are also linked to the HRO cluster in amphioxus [64], we suggest that the ancestral HRO cluster consisted of more PRD members, including at least *Gsc* and *Otx* (see also [26]). In addition, the LIM family gene *Isl* has been proposed to be part of an ancient PRD-LIM class Giga-cluster [26], which is consistent with our data to the extent that *Isl* is syntenic with the members of the PRD-class cluster in *M. hongkongensis* (Fig. 6).

## Conclusions

A high quality, chromosomal-scale genome assembly for the culturally, economically and ecologically important bivalve, the Hong Kong oyster (*Magallana hongkongensis*) is presented in this study, alongside insights into major patterns underlying genome evolution. Comparisons of the homeobox gene families of the Mega- and Giga-clusters imply that levels of inter-chromosomal rearrangements have been low in this oyster lineage relative to the bilaterian ancestor. Nevertheless, homeobox clusters such as Hox, ParaHox, NK and HRO, whilst still detectable to at least some extent, are undergoing varying degrees of dispersal, which has implications for the regulation of these genes and their roles during development. The genomic resources provided here also establish a foundation for scientifically-driven aquaculture development, as well as potentially important conservation tools for the species.

## Methods

### Sample collection and genome sequencing

Hong Kong oysters (*M. hongkongensis*) were collected from Lau Fau Shan in Deep Bay, Hong Kong, and samples for genome sequencing originate from a single individual (Fig. 1a). Genomic DNA (gDNA) was extracted using the PureLink Genomic DNA Mini Kit (Invitrogen) following the manufacturer’s protocol. Extracted gDNA was subjected to quality control using a Nanodrop spectrophotometer (Thermo Scientific) and gel electrophoresis. Qualifying samples were sent to Novogene, and Dovetail Genomics for library preparation and sequencing. Details of the sequencing data can be found in Supplementary information S1.

### Chicago and dovetail library preparation and sequencing

A Chicago library and a Dovetail HiC library were prepared as described previously [59]. Briefly, ~ 500 ng of high molecular weight genomic DNA (mean fragment length = 85 kbp) was reconstituted into chromatin in vitro and fixed with formaldehyde. Fixed chromatin was digested with DpnII, the 5′ overhangs filled in with biotinylated nucleotides, and free blunt ends were ligated. After ligation, crosslinks were reversed and the DNA purified from protein. Purified DNA was treated to remove biotin that was not internal to ligated fragments. The DNA was then sheared to ~ 350 bp mean fragment size and sequencing libraries were generated using NEBNext Ultra enzymes and Illumina-compatible adapters. Biotin-containing fragments were isolated using streptavidin beads before PCR enrichment of each library. The Chicago libraries were sequenced on an Illumina HiSeq X to produce 241 million 2 × 150 bp paired end reads, which provided 96.86 x physical coverage of the genome (1–100 kb pairs), while the Dovetail libraries were sequenced on an Illumina HiSeq X to produce 212 million 2 × 150 bp paired end reads, which provided 3885.16 x physical coverage of the genome (10–10,000 kb pairs).

### Genome assembly

Chromium WGS reads were separately used to make a de novo assembly using Supernova (v 2.1.1), specifying the parameter “--maxreads = 274,866,667” (raw coverage = 56.15x). The Supernova output pseudohap assembly, shotgun reads, Chicago library reads, and Dovetail HiC library reads were used as input data for HiRise, a software pipeline designed specifically for using proximity ligation data to scaffold genome assemblies [59]. An iterative analysis was conducted. First, Shotgun and Chicago library sequences were aligned to the draft input assembly using a modified SNAP read mapper (http://snap.cs.berkeley.edu). The separation of Chicago read pairs mapped within draft scaffolds were analyzed by HiRise to produce a likelihood model for genomic distance between read pairs, and the model was used to identify and break putative misjoins, to score prospective joins, and make joins above a threshold. After aligning and scaffolding Chicago data, Dovetail HiC library sequences were aligned and scaffolded following the same method. After scaffolding, shotgun sequences were used to close gaps between contigs.

### Gene model prediction

Raw sequencing reads from 13 transcriptomes were downloaded from the Sequence Read Archive (SRA) (SRR4035452, SRR4035451, SRR7777763, SRR7777764, SRR7777765, SRR7777766, SRR7777767, SRR7777768, SRR6201765, SRR1013751, SRR1013750, SRR949615 and SRR949616) and pre-processed with quality trimmed by trimmomatic (version 0.33, with parameters “ILLUMINACLIP:TruSeq3-SE.fa:2:30:10 SLIDINGWINDOW:4:5 LEADING:5 TRAILING:5 MINLEN:25”) [7]. For the nuclear genomes, the genome sequences were cleaned and masked by Funannotate (v1.6.0, https://github.com/nextgenusfs/funannotate) [60], the softmasked assembly were used to run “funannotate train” with parameters “--max_intronlen 350,000” to align RNA-seq data, ran Trinity [31], and then ran PASA [32]. The PASA gene models were used to train Augustus in “funannotate predict” step following manufacturers recommended options for eukaryotic genomes (https://funannotate.readthedocs.io/en/latest/tutorials.html#non-fungal-genomes-higher-eukaryotes). Briefly, the gene models were predicted by funannotate predict with parameters “--repeats2evm --protein_evidence uniprot_sprot.fasta --genemark_mode ET --busco_seed_species metazoa --optimize_augustus --busco_db metazoa --organism other --max_intronlen 350000”, the gene models from several prediction sources including ‘GeneMark(Lomsadze et al.)’: 71776, high-quality Augustus predictions (HiQ): 12511, ‘pas a[32]’: 22203, ‘Augustu s[72]’: 33008, ‘GlimmerHM M[50]’: 93209, ‘sna p[37]’: 147191 were passed to Evidence Modeler [32](EVM Weights: {‘GeneMark’: 1, ‘HiQ’: 2, ‘pasa’: 6, ‘proteins’: 1, ‘Augustus’: 1, ‘GlimmerHMM’: 1, ‘snap’: 1, ‘transcripts’: 1}) and generated the final annotation files, and then used PASA to update the EVM consensus predictions, added UTR annotations and models for alternatively spliced isoforms.

### Repetitive element annotation

Repetitive elements were identified using an in-house pipeline. First, elements were identified with RepeatMasker v4.1.0 [73] using the *mollusca* RepBase [35] repeat library. Low-complexity repeats and RNA were not masked (*−nolow* and *-norna*) and a sensitive (*−s*) search was performed. Following this, a de novo repeat library was constructed using RepeatModeler v1.0.11 [74], including RECON v1.08 [9] and RepeatScout v1.0.5 [62]. Novel repeats identified by RepeatModeler were analysed using a ‘BLAST, Extract, Extend’ process [63]. Briefly, up to the top 40 hits for each TE family identified by RepeatModeler were retained from a BLASTn search against the genome [13]. Sequences were extracted together with 1000 base pairs of flanking sequence at each end. Each set of family sequences were aligned using MAFFT [38]. Alignments were then trimmed with trimAl [14] to retain high-quality positions in the alignment (*−gt* 0.6 *-cons* 60). New consensus sequences were then computed with EMBOSS [67] cons (−*plurality* 3) to generate a new TE library with extended consensus sequences. This process was repeated through 5 iterations to obtain maximum-length consensus sequences. The resulting de novo repeat library was utilised to identify repetitive elements using RepeatMasker. In addition to the parameters stated above, the final RepeatMasker score threshold was set at the more conservative level of 400 (*−cutoff* 400) to exclude poor matches unlikely to be true TE sequences. Additionally, following this, all repeats less than 100 bp in length were also removed before the final element quantification to further improve the quality of the final repeat annotation. All plots were generated using Rstudio v1.2.1335 [70, 83] with R v3.5.1 [84] and ggplot2 v3.2.1 [87].

### Gene family annotation and tree building

Potential homeobox genes were first identified by similarity searches using homeodomain sequences from *C. gigas* ([69], [5]), *B. floridae* and *T. castaneum* retrieved from HomeoDB [96], and retrieved from the genome and transcriptomes using tBLASTn [1] in *M. hongkongensis* and all published mollusc genomes (Table 1). NCBI CD-search [48] was further used to validate the presence of homeodomains in the retrieved sequences. Identity of each putative gene was then tested by comparison to sequences in the NCBI nr database using BLASTx and BLASTp along with phylogenetic analyses. For phylogenetic analyses of gene families, DNA sequences were translated into amino acid sequences and aligned to other members of the gene family and phylogenetic trees were constructed using MEGA [40] and assigned homology based on a previous study on lophotrochozoan homeobox genes [5].

## Supplementary information

**Additional file 1. **Sequencing data of oyster *M. hongkongensis* generated in this study.

**Additional file 2.** Estimated repeat content present in the genome for the Pacific oyster, Sydney rock oyster, and Hong Kong oyster for both the assembly presented here, and the assembly of Peng et al. [46].

**Additional file 3.** Repeat landscape plots.

**Additional file 4.** Homeobox gene sequences and genomic locations in mollusc genomes.

**Additional file 5.** Homeobox gene trees constructed with Maximum-likelihood method (LG + G) based on the homeodomain sequences (1000 bootstraps).

**Additional file 6.** TALE-class homeobox gene trees constructed with Maximum-likelihood method (LG + G + I) based on the homeodomain sequences (1000 bootstraps).

## Data Availability

The final chromosome assembly was submitted to NCBI Assembly under accession number WFKH00000000 in NCBI. The raw reads generated in this study have been deposited to the NCBI database under the BioProject accessions: PRJNA576886, the genome annotation files were deposited in the Figshare 10.6084/m9.figshare.12715490.v1.
