# Supplementary material for: Reconstruction of ancient homeobox gene linkages inferred from a new high-quality assembly of the Hong Kong oyster (Magallana hongkongensis) genome
Source: BMC Genomics. 2020 Oct 15;21:713. doi: 10.1186/s12864-020-07027-6 (PMC7566022; doi:10.1186/s12864-020-07027-6)
Supplement: Supplementary file 3 — Additional file 3. Repeat landscape plots. [file 12864_2020_7027_MOESM3_ESM.zip › S3. TE/magHon.hui_RL.html]

Interspersed Repeat Landscape

Interspersed Repeat Landscape


---

© RepeatMasker.org
