# Supplementary material for: Reconstruction of ancient homeobox gene linkages inferred from a new high-quality assembly of the Hong Kong oyster (Magallana hongkongensis) genome
Source: BMC Genomics. 2020 Oct 15;21:713. doi: 10.1186/s12864-020-07027-6 (PMC7566022; doi:10.1186/s12864-020-07027-6)
Supplement: Supplementary file 5 — Additional file 5. Homeobox gene trees constructed with Maximum-likelihood method (LG + G) based on the homeodomain sequences (1000 bootstraps). [file 12864_2020_7027_MOESM5_ESM.pptx]

## Slide 1
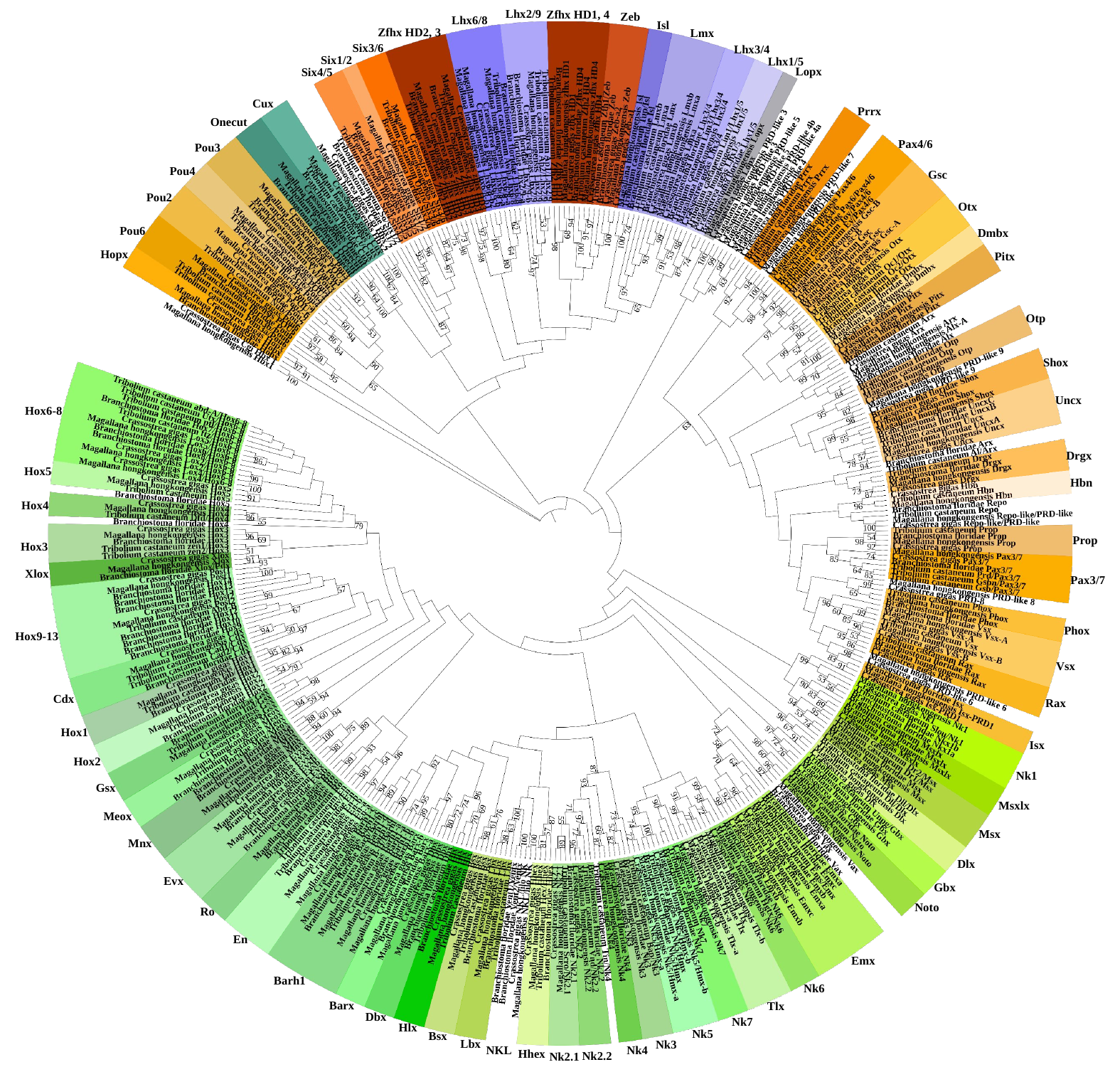

Lhx2/9
Zfhx HD1, 4
Zeb
Lhx6/8
Isl
Zfhx HD2, 3
Lmx
Six3/6
Lhx3/4
Six1/2
Lhx1/5
Lopx
Six4/5
Cux
Prrx
Onecut
Pax4/6
Pou3
Pou4
Gsc
Pou2
Otx
Pou6
Dmbx
Hopx
Pitx
Otp
Shox
Uncx
Hox6-8
Drgx
Hox5
Hbn
Hox4
Prop
Hox3
Xlox
Pax3/7
Phox
Hox9-13
Vsx
Cdx
Rax
Hox1
Isx
Hox2
Nk1
Gsx
Msxlx
Meox
Msx
Mnx
Dlx
Evx
Gbx
Noto
Ro
En
Emx
Barh1
Nk6
Barx
Tlx
Dbx
Nk7
Hlx
Nk5
Bsx
Lbx
Nk3
NKL
Nk4
Hhex
Nk2.2
Nk2.1
