# Supplementary figures and images for: Reconstruction of ancient homeobox gene linkages inferred from a new high-quality assembly of the Hong Kong oyster (Magallana hongkongensis) genome
Source: BMC Genomics. 2020 Oct 15;21:713. doi: 10.1186/s12864-020-07027-6 (PMC7566022; doi:10.1186/s12864-020-07027-6)

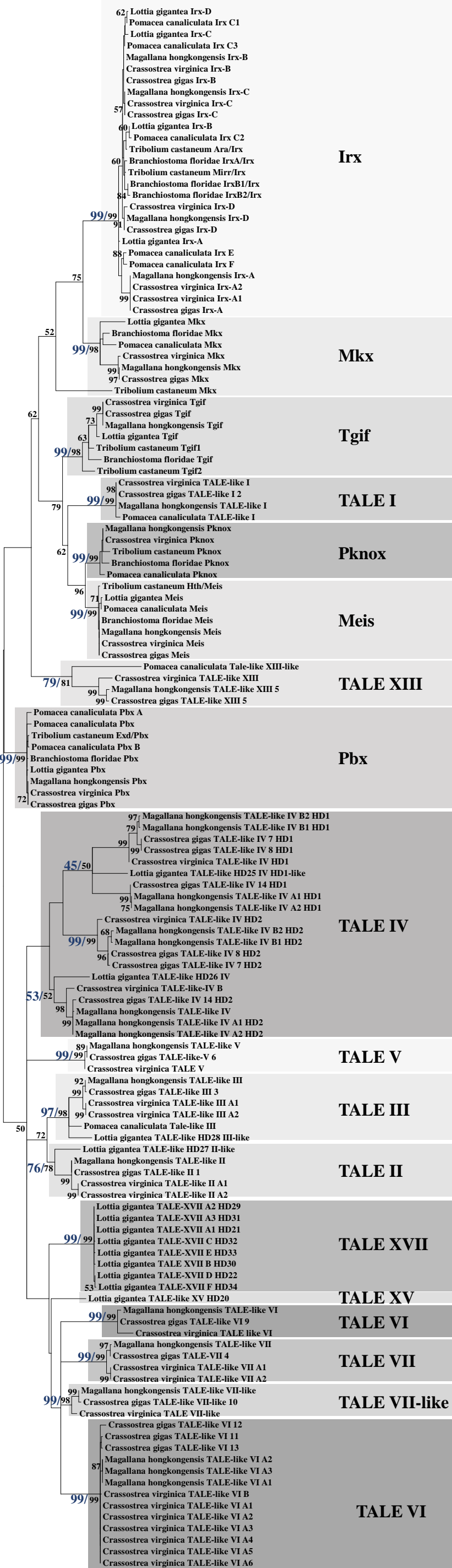

Supplement: Supplementary file 6 — Additional file 6. TALE-class homeobox gene trees constructed with Maximum-likelihood method (LG + G + I) based on the homeodomain sequences (1000 bootstraps). [file 12864_2020_7027_MOESM6_ESM.pdf]
